# Supplementary material for: Stress Induces Trait Variability across Multiple Spatial Scales in the Arid Annual Plant Anastatica hierochuntica
Source: Plants (Basel). 2024 Jan 16;13(2):256. doi: 10.3390/plants13020256 (PMC10820187; doi:10.3390/plants13020256)
Supplement: Supplementary file 1 [file plants-13-00256-s001.zip › plants-2737719-supplementary.pdf]

Supplementary Materials

Stress Induces Trait Variability across Multiple Spatial Scales in the Arid Annual Plant *Anastatica hierochuntica*

Nir Krintza [1,†], Efrat Dener [2,†] and Merav Seifan [2,*]

[1] Albert Katz International School for Desert Studies, Jacob Blaustein Institutes for Desert Research, Ben-Gurion University of the Negev, 8499000, Israel; nir.krintza@gmail.com
[2] Mitrani Department of Desert Ecology, Swiss Institute for Dryland Environment and Energy Research, Jacob Blaustein Institutes for Desert Research, Ben-Gurion University of the Negev, 849900, Israel; efratde@gmail.com
[*] Correspondence: seifan@bgu.ac.il
[†] These authors contributed equally to this work.

Field supplementary material:

Figure S1: results for testing the potential effects of between site (aridity index) and within site (soil salinity, local elevation) conditions on trait values of *A. hierochuntica* individuals (GLMM model, Fig. 1, Table 2 main text). None of the results are significant.

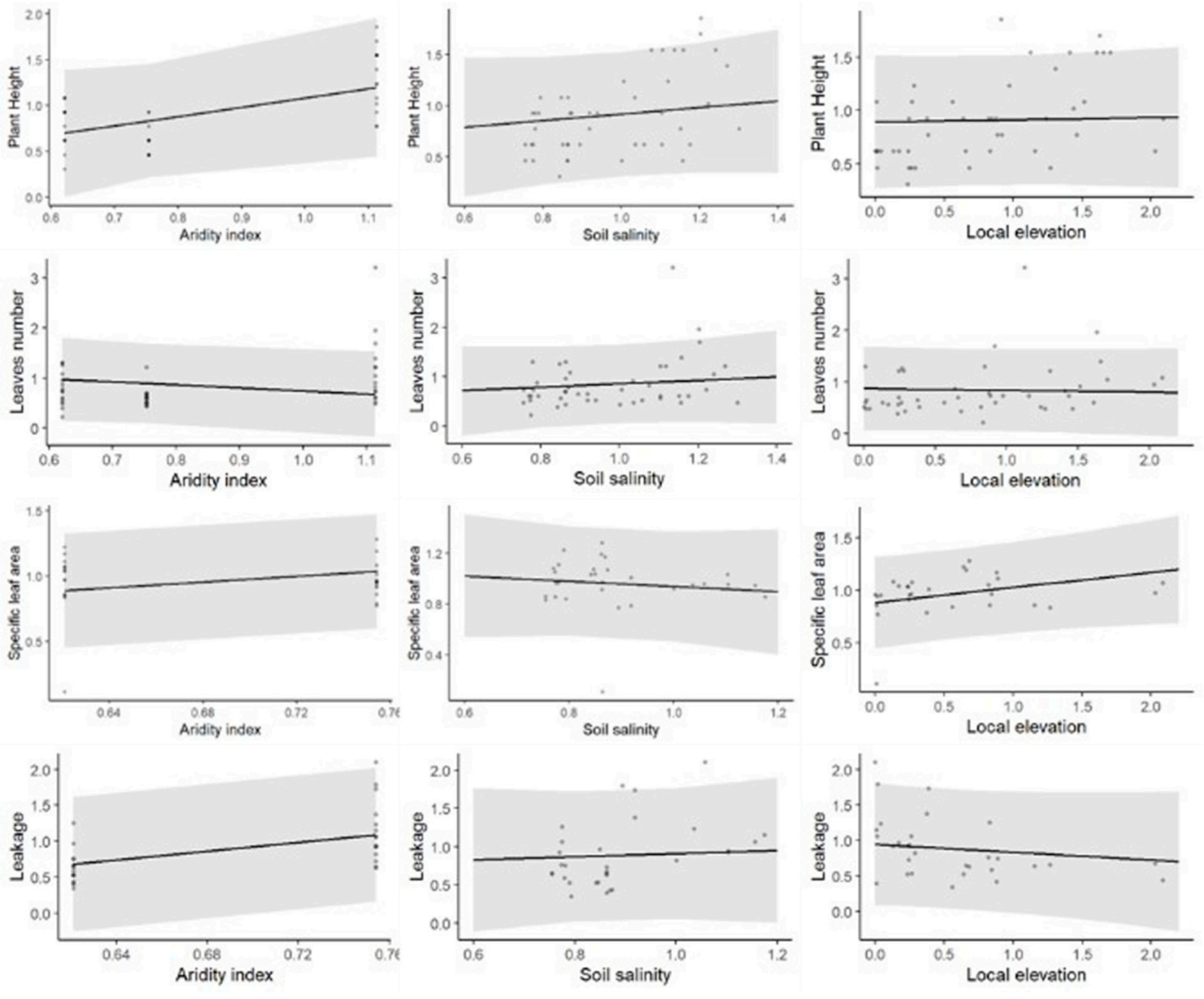

Net house supplementary material:

Full results of irrigation experiment:

		Plant height	*Leaves number*	*SLA*	*Plant biomass*	*Fruits number*
Treatment		**4.2362 ***	0.0033	0.1488	**70.5214 *****	0.0061
Site scale	Aridity	1.9558	1.5669	**7.9859 ****	1.3120	0.3517
	Treatment * Aridity	3.0398	**6.6812 ****	**9.2439 ****	1.9603	0.8697
Local scale	Salinity	0.7888	0.0064	0.9785	1.7874	0.0690
	Local elevation	0.7480	1.8848	0.7049	0.2212	0.1405
	Treatment * Salinity	**5.6268 ***	**4.0766 ***	1.3001	**3.8442 ***	1.0188
	Treatment * Local elevation	0.7832	0.7389	2.3965	0.3030	0.0978
Conditional R^2		0.313	0.248	0.711	0.586	0.026

Table S1: GLMM results (χ2 with 1 df) for trait values in the net house experiment using seeds from the plants sampled in the field analysis. Habitat conditions represent the conditions in which the mother plants were growing. Treatment represents the irrigation experiment. * represents 0.05< P value <0.01, ** represents 0.01< P-value <0.001, *** represents P-value < 0.001

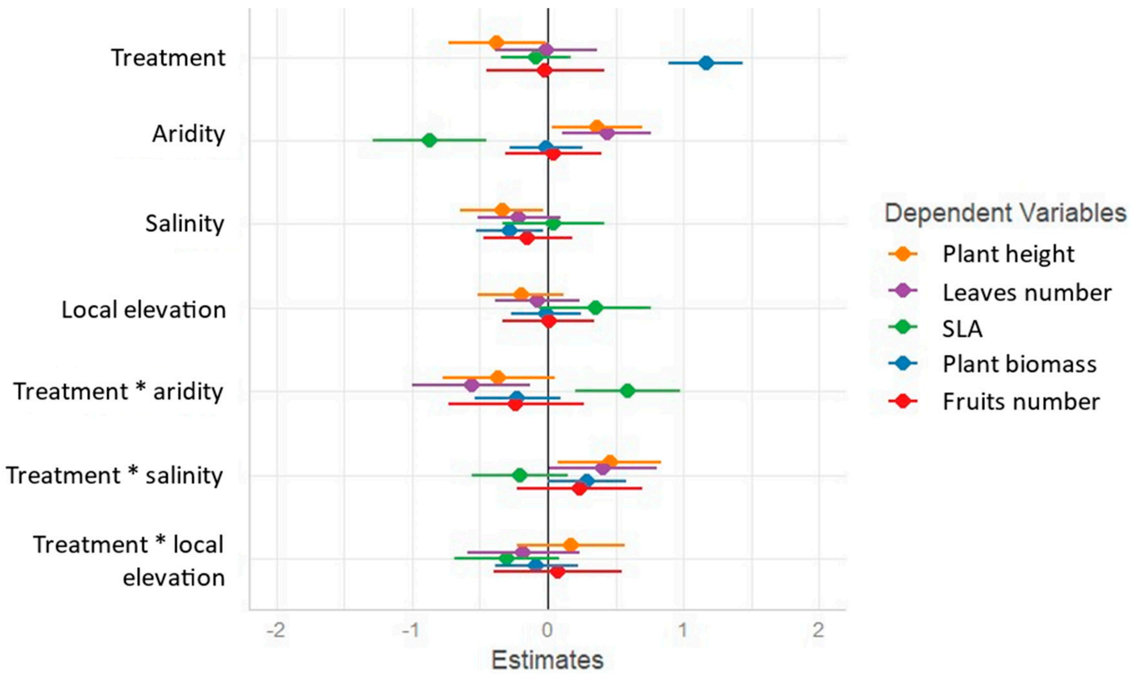

Figure S2: GLMM slope values (±SE) for the effects of irrigation treatment and between and within site conditions of the mother plants on trait of *A. hieochuntica* individuals.

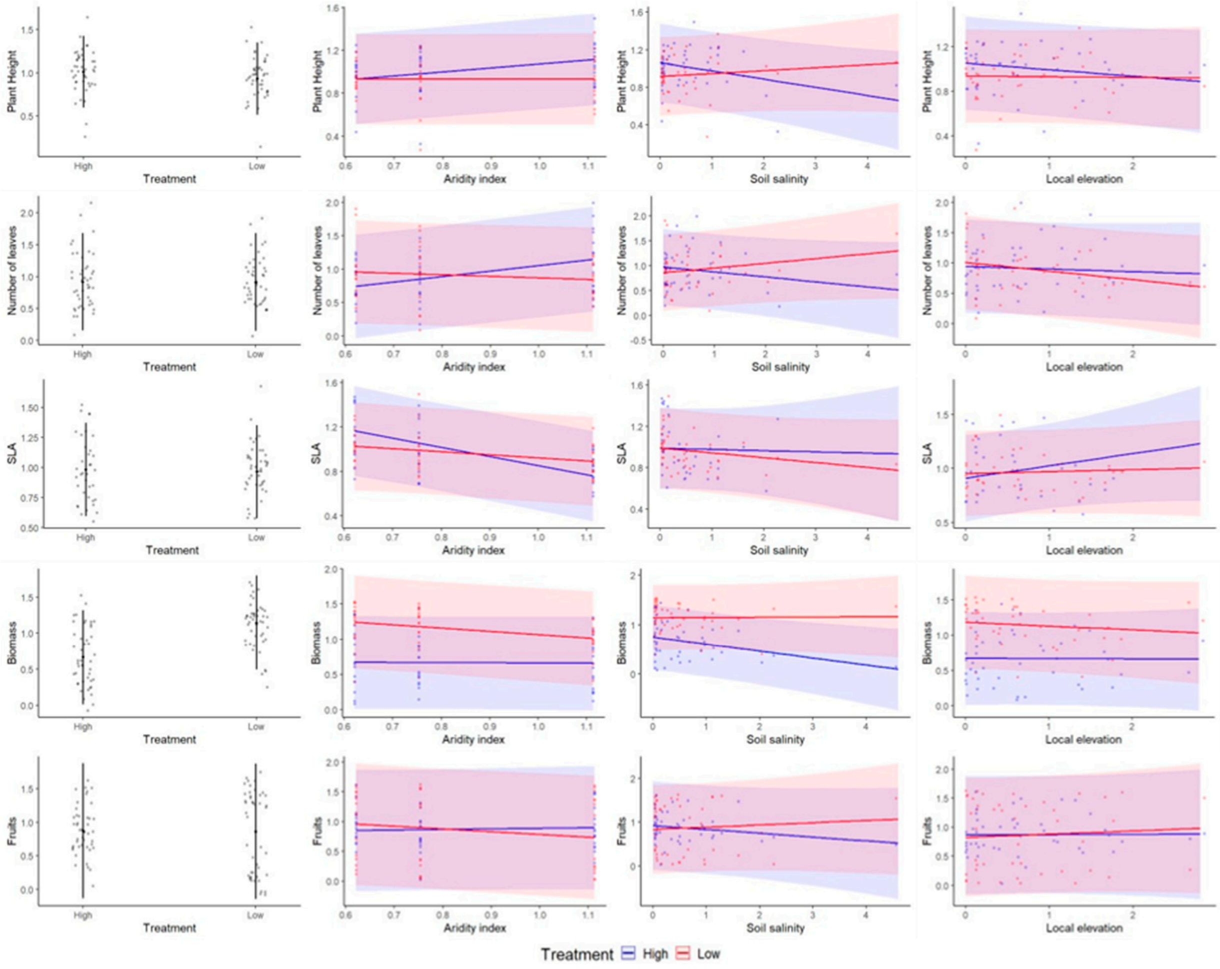

Figure S3: results of the effects of the irrigation treatment and the environmental conditions of the mother plants on traits of *A. hieochuntica* individuals. Colored background represents the confidence interval around the mean value of the irrigation treatment.

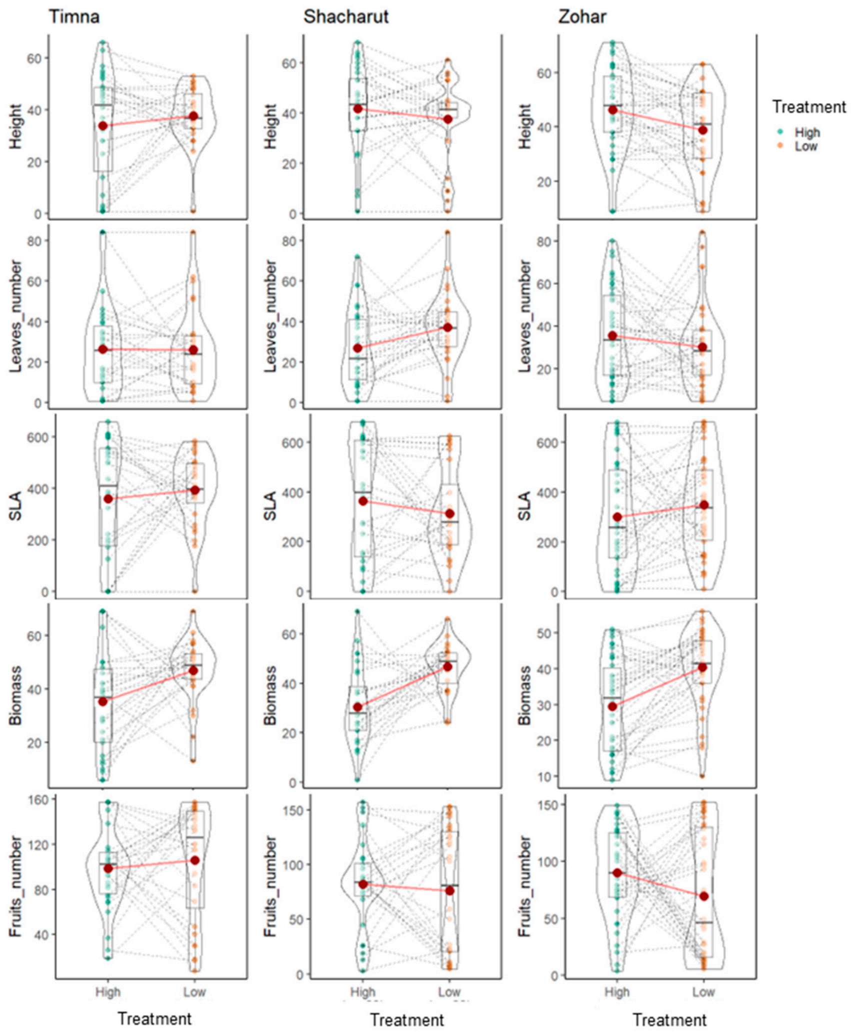

Figure S4: Reaction norms of plant responses, based on the five traits measured, to irrigation treatments (high and low). Plants are grouped in the figure according the population of origin and organized according to the aridity gradient, with Timna the most arid site and Zohar the least arid (see site details in table 1 in the main text).
